# 成纤维生长因子受体在非小细胞肺癌中的研究进展

**DOI:** 10.3779/j.issn.1009-3419.2013.11.10

**Published:** 2013-11-20

**Authors:** 丹 蒲, 梅 侯

**Affiliations:** 610041 成都，四川大学华西医院肿瘤中心 Cancer Center, West China Hospital, Sichuan University, Chengdu 610041, China

**Keywords:** 非小细胞肺癌, 成纤维生长因子受体, 表皮生长因子受体, 抗血管生成治疗, Lung neoplsms, Fibroblast growth factor receptor, Epidermal growth factor receptor, Anti-angiogenesis treatment

## Abstract

肺癌严重威胁人类的健康，近年来肺腺癌的治疗方式取得了巨大的进展，许多靶向治疗药物已广泛应用于临床并不同程度的使患者受益。而肺鳞癌中表皮生长因子受体（epidermal growth factor receptor, *EGFR*）基因突变以及*ALK*融合基因发生率低，使用吉非替尼及克唑替尼等酪氨酸激酶抑制剂治疗有效率低。一直以来成纤维生长因子（fibroblast growth factor, FGF）通路的异常被认为与肿瘤的增殖、血管生成等方面密切相关，近年越来越多的研究发现非小细胞肺癌中存在成纤维生长因子受体1（fibroblast growth factor receptor 1, *FGFR1*）基因扩增，体外及体内实验发现阻断FGF通路可降低肿瘤细胞的增殖、抑制远处转移、逆转靶向治疗耐药。本文就FGFR在非小细胞肺癌中的表达以及作为治疗靶点的前景作一综述。

## Abstract (en)

Lung cancer is severely threatening human health. In recent years, the treatment for lung adenocarcinoma has made a great progress, targeted therapy has been widely applied in clinic, and benefits amount of patients. However, in squamous cell lung cancer, the incidence of epidermal growth factor receptor (*EGFR*) gene mutant and *ALK* fusion gene are low, and targeted therapy like Tarceva and crizotinib, can hardly work. Since the fibroblast growth factors (fibroblast growth factor, FGF) pathway is considered to be related to tumor cell proliferation, metastasis and angiogenesis, more and more researches proved the amplification of fibroblast growth factor receptor (FGFR) in squamous cell lung cancer. Experiments *in vivo* and *in vitro* found that blocking FGF pathway could reduce the proliferation of tumor cells and inhibit metastasis. The FGF pathway might be a new target for treatment of squamous cell lung cancer. This article reviews the effect of FGFR in tumorigenesis, as well as the prospect as a therapeutic target in non-small cell lung cancer.

肺癌是最常见的恶性肿瘤之一，在2008年全球约137万人死于肺癌。肺癌患者中约85%为非小细胞肺癌（non-small cell lung cancer, NSCLC），研究人员在肺腺癌中发现表皮生长因子受体（epidermal growth factor receptor, *EGFR*）基因突变，*ALK*融合基因等，将其作为靶点进行分子靶向治疗，取得了令人瞩目的成果，使部分肺腺癌患者的生存质量和总生存期得到了明显的改善。然而，*EGFR*基因突变及*ALK*融合基因在肺鳞癌中少见，以其为靶点的治疗措施在肺鳞癌患者中难以奏效。2012年美国癌症基因图谱协作网（The Cancer Genome Atlas Research Network）通过对178例肺鳞癌组织的分析，描绘出了肺鳞癌大致的基因及表观遗传学改变，发现*EGFR*及*K-ras*突变在鳞癌中十分少见，而更为常见的是成纤维生长因子受体（fibroblast growth factor receptor, FGFR）激酶家族的异常，其中包括成纤维生长因子受体1基因扩增^[1]^。FGF信号通路介导NSCLC的生长和血管生成，同时被认为与抗血管内皮生长因子（vascular endothelial growth factor, VEGF）及抗EGFR治疗耐药相关。FGFR1作为FGF家族的酪氨酸激酶受体，其基因扩增被认为可作为非小细胞肺癌治疗的分子靶点。

## 1 FGF信号通路

FGF与FGFR普遍存在于正常细胞中，共同构成FGF信号通路，在细胞生长、分化、迁移、血管生成以及组织创伤修复中起重要作用，近年研究表明，该通路在肿瘤的发生、发展过程中也扮演重要角色。FGF信号通路至少由22种FGFs和4种FGFR构成。FGFs是一类多肽生长因子，除了FGF19、FGF21、FGF23可通过类似激素的内分泌途径入血，大部分FGFs通过自分泌或旁分泌途径起生理作用。FGFs在细胞膜上主要存在两类受体，一类是低亲和力受体，即肝素硫酸蛋白多糖（heparin sulfate proteoglycan, HSPG），细胞外的FGFs通过与HSPG结合协助FGFRs发生二聚化、自磷酸化以及细胞内途径的激活，同时使FGFs免于降解。另一类是高亲和力受体，即FGFR，属于跨膜酪氨酸激酶受体家族（transmembrane receptor tyrosine kinase, RTK），由*FGFR1*、*FGFR2*、*FGFR3*、*FGFR4*基因编码，由胞外区，跨膜区及具有酪氨酸激酶活性的胞内区构成。胞外区包括配体结合位点、酸盒及2个或3个免疫球蛋白样结构域（Ⅰg Ⅰ, Ⅰg Ⅱ, Ⅰg Ⅲ），如图 1。Ⅰg Ⅰ被认为与受体抑制相关^[2]^，而不参与配体的特异性结合，缺乏Ⅰg Ⅰ并不影响FGFRs与FGFs的亲和性，Ⅰg Ⅲ与Ⅰg Ⅱ是FGFRs与配体结合的部位，并决定结合的特异性。由于mRNA剪切形式的差异，Ⅰg Ⅲ存在Ⅲb和Ⅲc两种异构体，不同的FGFs其优势结合位点不同^[3]^，如表 1，FGF7和FGF1倾向于结合FGFR Ⅲb，其余的则对FGFR Ⅲc具有更好的亲和性。在组织中，FGFR的类型间具有一定的分布特征，Ⅲc异构体常见于间质组织中，而Ⅲb异构体最多见于上皮细胞中，间质组织需要的配体常常通过邻近上皮组织分泌，而上皮组织需要的配体常常通过邻近间质组织分泌，这可能是维持细胞微环境稳定的一种方式，其异常可能是导致肿瘤发生以及肿瘤细胞上皮间质转化的一个原因。当FGFR与相应配体结合后发生构型改变，使得胞内段酪氨酸激酶残基磷酸化，活化的酪氨酸激酶残基作为与下游信号通路的接驳点，可激活多条下游信号通路产生生物学效应（图 1），例如，FGFR近膜部的酪氨酸激酶磷酸化位点与接连蛋白FRS2结合后，使得FRS2的多个酪氨酸残基磷酸化，招募SOS、GRB2、GAB1蛋白形成复合物激活Ras-Raf-MapK、PI3K信号通路；FGFR的-COOH端的酪氨酸激酶磷酸化位点与SH2结合，导致PLCγ磷酸化，促进细胞内钙离子释放，激活PKC。另外，根据细胞微环境的差异，FGFR还可激活包括Shb、Src、Crk、RSK在内的其他途径，但目前研究表明，Ras-Raf-MapK、PI3K-Akt、Stats和PLCγ是FGFR的四个主要的下游信号通路。

**1:** 22种FGFs的7个亚型及其特异性受体 The subfamilies of FGFs and their specific receptors

Receptor isoform	Ligand	Ligand subfamily
FGFR Ⅲb	FGF1 subfamily	FGF1, FGF2
	FGF7 subfamily	FGF3, FGF7, FGF10, FGF22
	FGF4 subfamily	FGF4, FGF5, FGF6
	FGF9 subfamily	FGF9, FGF16, FGF20
FGFR Ⅲc	FGF8 subfamily	FGF8, FGF17, FGF18
	FGF11 subfamily	FGF11, FGF12, FGF13, FGF14
	FGF19 subfamily	FGF19, FGF21, FGF23

**1:**
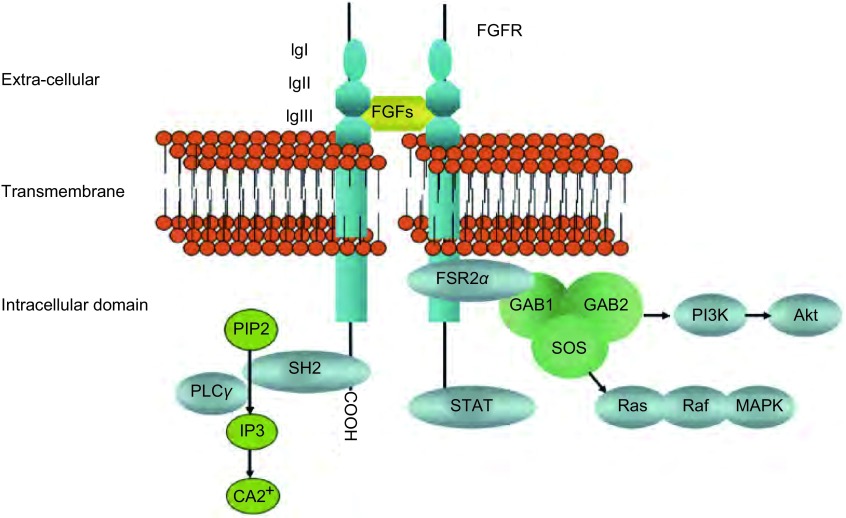
成纤维生长因子受体的结构及其下游信号通路 The structure and downstream signaling pathways of fibroblast growth factor receptor. PIP2: phosphatidylinosital-4, 5-biphosphate; PLC*γ*: phospholipase C*γ*; IP3: inositol triphosphate; FSR2*α*: FGFR substrate 2*α*; GRB2: growth factor receptor bound 2; GAB1: Grb2-associated binder; SOS: son of sevenless; MAPK: mitogen-activated protein kinases; STAT: signal transducers and activators of transcription; PI3K: phosphatidylinositol 3-hydroxy kinase; Akt: serine-threonine protein kinase.

## 2 NSCLC中FGFR的表达

FGFR的高表达在包括前列腺、乳腺、胃、口腔等多种肿瘤中均有发现^[4-7]^。1999年，Berger ^[8]^等在NSCLC中发现FGF2、FGFR的表达升高后，陆续多个研究证实非小细胞肺癌中存在*FGFR*基因异常^[9-11]^，包括染色体异位、基因扩增、点突变、修复和剪切异常等^[12]^，常用的检测手段为免疫组化、荧光原位杂交以及RT-PCR。

*FGFR1*基因位于8号染色体短臂，Zhang等^[13]^报道，中国人中*FGFR1*基因扩增率在肺鳞癌中为12.5%（6/48），肺腺癌中为7%（5/76），Heist等^[10]^通过分析226例美国患者后显示肺鳞癌中FGFR1扩增率为16%，在德国的一项研究中10.5%的肺鳞癌以及4.7%的肺腺癌患者中存在*FGFR*基因扩增^[11]^。结合目前中外研究显示，肺鳞癌中FGFR1扩增率为10%-20%，明显高于肺腺癌，且亚欧人群中并不存在明显的种族差异。Heist的研究^[10]^同时也指出*FGFR*基因扩增与年龄、性别、肿瘤分期以及吸烟史无相关性，*FGFR*基因状态不同的人群间总生存期无统计学差异，这与Weiss之前的研究结论不一致，Weiss等^[14]^等通过分析232例NSCLC患者，其结果显示，*FGFR1*基因扩增主要见于吸烟者，且*FGFR1*基因扩增的患者倾向于不良的生存预后。Tran等^[15]^的研究中从未吸烟的患者虽然例数较少，但全部为FGFR1阴性，支持Weiss的研究结论，不同的是，该研究的多因素分析中*FGFR1*基因拷贝数高的患者倾向于更长的总生存期（overall survival, OS），研究者认为*FGFR1*基因拷贝数升高是NSCLC有益的预后预测因子。既往研究结论不一致，有可能是受样本量及分层因素的影响，包括病理类型、是否手术以及*EGFR*基因状态等，例如，Kohler等^[11]^一个样本量非常小的分析提示FGFR1扩增的肺腺癌倾向于有生存获益，假如这种现象并非偶然，那么如果将腺癌与鳞癌共同纳入分析就有可能得出NSCLC中FGFR1扩增倾向于生存获益的结论；另外*EGFR*基因突变的患者在后续治疗中使用酪氨酸激酶抑制剂（tyrosine kinase inhibitor, TKI）类药物可能导致OS无差别。近期韩国研究者Kim等^[16]^的研究纳入262例肺鳞癌根治术后患者，研究同时检测了*EGFR*及*KRAS*基因突变，结果显示*FGFR1*基因高度扩增的患者较*FGFR*基因无扩增或低度扩增的患者，其无疾病生存期（disease-free survival, DFS）及总生存期OS明显较短（DFS：26.9个月*vs* 94.6个月，*P* < 0.001；OS：51.2个月*vs* 115.0个月，*P* < 0.002），吸烟患者的FGFR1扩增率明显高于已戒烟者和从不吸烟的患者（28.9% *vs* 2.5% *vs* 0），与以往研究一致，*FGFR1*基因状态与性别、肿瘤分期、脉管浸润、胸膜受侵等无明显相关性，表明*FGFR1*基因扩增预示鳞癌术后患者的预后不良，且不存在明显的“优势人群”，在选择FGFR抑制剂时需要对患者进行*FGFR*基因检测。

FGFR2与FGFR1结构相似，其在膀胱癌中的表达降低，而增加FGFR2表达可阻断膀胱癌细胞株的生长^[17]^，因此一些研究者认为FGFR2对肿瘤有抑制作用。在乳腺癌移植瘤模型中^[18]^，阻断FGFR1肿瘤生长明显放缓，然而阻断FGFR2可使FGFR1表达上调，导致肿瘤长大以及肿瘤血管生成，研究者认为FGFR1密切参与肿瘤形成，而FGFR2并不直接参与肿瘤增殖，反而可能存在抑制作用。但FGFR2远不能被笼统的看作是抑癌基因，因为除了在部分生殖泌尿系统肿瘤中发现FGFR2表达降低之外，在胃癌、胰腺癌、乳腺癌、肺癌中都发现FGFR2的上调，其表达及作用在不同肿瘤中产生差异的原因尚不清楚，可能与肿瘤微环境相关。另外，Sasaki等^[19]^在日本肺癌人群，以及Matakidou等^[20]^在欧洲肺癌人群中没有发现*FGFR4*的突变，但*FGFR4* Gly388Arg存在基因多态性，目前研究显示其基因型与吸烟状态、病理分型、分期以及OS没有相关性。虽然Sasaki的研究显示Gly388Arg的基因多态性与术后淋巴结阳性患者的不良OS相关，但尚不足以证明*FGFR4* Gly388Arg的基因多态性可作为肺癌预后的预测因子。

FGF信号通路被认为可作为肺鳞癌靶向治疗的潜在靶点，*FGFR1*基因扩增见于约20%的肺鳞癌患者中，在体内及体外试验中，FGFR抑制剂可使存在*FGFR1*基因扩增的肿瘤体积明显缩小，这些似乎都预示着FGFR1在肺鳞癌中的靶向治疗前景，目前关于FGFR在肺癌中的研究也主要集中在FGFR1。

## 3 FGFR与NSCLC的治疗

### 3.1 FGFR与抗血管生成治疗

肿瘤新生血管是肿瘤生长的营养支持以及远处转移的重要途径，目前抗血管生成的靶向治疗药物主要作用于VEGF通路，但Bevacizumab（VEGF单克隆抗体）与化疗联用并不能使患者OS得到明显的延长^[21]^，原因是大部分患者的获益是短暂的，还有部分患者不能从加用Bevacizumab的治疗中获益。抗血管生成药物的作用靶点在血管内皮细胞，这类细胞并不像肿瘤细胞一样具有无限增殖的能力，因此对Bevacizumab的耐药并不倾向于VEGF信号通路中某些位点的基因突变，而更可能来源于肿瘤微环境或者其它替代途径的改变。FGFs是最先被发现的血管生长因子之一，它们在内皮细胞增殖、迁移、细胞黏附等血管形成过程中的作用已被广泛研究。体外实验中，FGFR1、FGFR2与配体FGFs1、FGFs2、FGFs4、FGFs8结合后通过MAPK、PKC信号传导通路促进上皮细胞增殖，与配体FGFs1、FGFs2、FGFs8、FGFs10结合后可通过刺激上皮细胞的趋化性，促进上皮细胞的迁移，同时，FGF1、FGFs2、FGFs4还可上调纤溶酶原激活物和基质金属蛋白酶促进细胞外基质的改变^[22]^，为新生血管的形成提供适宜的条件，FGF2、FGFs8可诱导上皮细胞形成毛细血管样结构，在血管的构型和成熟方面起重要作用^[23]^。有足够的证据表明FGFs与VEGF通路在促进血管生成中具有协同作用，FGF2可上调内皮细胞中VEGFR和VEGF的表达，VEGF也可促进FGF2表达[24.25]，阻断VEGF后FGF2表达减少，反过来，阻断FGFR1、FGFR2的表达同样可降低VEGF水平^[26]^。同时，FGF介导的血管生成并不完全依赖于VEGF，在VEGF高表达的情况下，FGF-2过度表达仍能大大增加新生血管形成。Ogawa等^[27]^在多个肺癌细胞株中发现，阻断FGFR1能有效抑制抗VEGF耐药的肿瘤血管生成，增加细胞凋亡，并且同时阻断FGFR1和VEGFR对肿瘤的抑制作用较单用其中一种更加明显。这表明FGF信号通路与VEGF信号通路在促进肿瘤血管生成中相互促进而又互不依赖，FGF通路可能是肿瘤逃避抗VEGF治疗的一种途径，预示着使用多靶点的抗血管生成治疗药物可能将是一个行之有效的方法。

### 3.2 FGFR与EGFR-TKI耐药

EGFR TKI在*EGFR*基因突变的NSCLC患者中的有效率高达70%，但部分患者存在原发耐药，另外几乎所有初始使用有效的患者随着用药时间的延长将会产生继发耐药。约50%的EGFR TKI耐药与EGFR T790M突变相关，5%-15%与Met扩增相关。另外仍有约30%原因不明，推测与酪氨酸激酶旁路途径的建立与激活相关，可能部分来自于*FGFR*基因改变。Terai等在吉非替尼耐药的NSCLC细胞株（PC9 GR）中发现肿瘤细胞的扩增通过依赖bFGF-FGFR1的途径，并且在PD173074（FGFR抑制剂）处理后可恢复对吉非替尼的敏感性。Ware等^[28]^的研究支持了Terai^[29]^的结论，他们在研究中培育了吉非替尼继发耐药的肺癌细胞株HCC4006，发现b-FGF和FGFR1的mRNA分别增加了2.5倍和9倍，联用AZD4547（FGFR抑制剂）可抑制耐药株的生长，他们同时发现耐药株中上皮型E-钙粘蛋白、交联蛋白表达降低，波形蛋白、N-钙粘蛋白、ZEB1、ZEB2等间质组织标记物升高，并且这种情况并不是偶然，在实验诱导的其他耐药株中也发现了近似的现象。目前在许多肿瘤类型中的研究显示，FGF信号通路可诱导细胞上皮间质转化（epithelial-mesenchymal transition, EMT），异常的FGFR1促进肿瘤细胞发生EMT的主要作用点在于激活Snail家族，MAPK信号通路可上调snail mRNA水平，PI3K/Akt信号通路可阻断抑制蛋白GSK-3β使Snail稳定表达，促进上皮间质转化，而具有间质表型的NSCLC对EGFR TKI的敏感性降低^[30]^。多数研究者认为吉非替尼的获得性耐药与FGF旁路信号通路的建立及其伴随的上皮间质转化相关，推测可能存在这样的一个模式：上皮表型为主的肿瘤细胞耐药与IGF1活化、Met扩增、EGFR T790M突变关系更为密切，而间质表型为主的肿瘤细胞耐药与PDGFR、FGFR信号通路相关。目前临床上，尚无条件分析患者使用EGFR TKI类药物前后肿瘤细胞表型及FGFR状态的改变，但在体外和动物实验中，FGF信号通路作为EGFR TKI治疗耐药的旁路途径已得到证实。目前一项厄洛替尼联合dovitinib（FGFR及VEGFR抑制剂）应用于晚期NSCLC患者的Ⅰ期临床试验（NCT01515969）正在进行，研究者期望联用FGFR抑制剂可对抗或延缓EGFR TKI耐药。

## 4 FGFR抑制剂在NSCLC中的应用

近年，多项FGFR酪氨酸激酶抑制剂已进入临床研究。Cediranib是一种多靶点口服酪氨酸激酶抑制剂，其靶点包括VEGFR、血小板衍生生长因子受体（platelet-derived growth factor receptor, PDGFR）、c-kit以及FGFR1，Cediranib联合PC方案（紫杉醇加卡铂）用于NSCLC患者的一项Ⅱ期-Ⅲ期临床研究显示，cediranib组的有效率提高（38% *vs* 16%, *P* < 0.001）^[31]^，虽然由于严重的毒副反应使得研究提前终止，但该研究展示出多靶点药物的应用前景，目前正在探索更低剂量的cediranib联合化疗的耐受性（NCT00795340）。BGJ398是选择性的FGFR酪氨酸激酶抑制剂，正在进行Ⅰ期临床试验（NCT01004224），试验针对包括肺癌在内的实体肿瘤，要求入组的患者必须存在*FGFR1*或*FGFR2*基因扩增，或是*FGFR3*突变。FGFR抑制剂的出现以及进一步研究，为肺癌尤其是肺鳞状细胞癌的治疗提供了新的选择。虽然目前开展临床试验（Brivanib, Pazopanib, XL999, E-3810, AZD4547, PD173074）的多种FGFR酪氨酸激酶抑制剂，同时也是VEGFR抑制剂，它们对肿瘤的抑制作用有可能首先来源于抑制新生血管形成，但有研究显示FGFR抑制剂本身还存在除抑制肿瘤血管生成之外的直接的抗肿瘤效应，该效应可能与凋亡因子caspase3活化相关，且FGF通路参与VEGFR抑制剂耐药，因此多靶点的酪氨酸激酶抑制剂仍有可能提高疗效或延长疾病进展时间。

FGF信号通路与肿瘤的发生及肿瘤血管形成密切相关，新近研究表明，它参与抗VEGF以及抗EGFR靶向治疗耐药。*FGFR1*基因扩增在肺鳞癌以及肺腺癌中均有发生，但在鳞癌中明显高于腺癌，在具有*FGFR1*基因扩增的肿瘤中使用FGFR抑制剂可增加肿瘤细胞凋亡，使肿瘤明显缩小，同时FGFR可解救抗VEGF和抗EGFR治疗耐药，发挥抗肿瘤效应。在NSCLC中，研究针对FGFR的靶向治疗药物具有重要的应用前景。
